# Exposure to dust and respiratory health among Australian miners

**DOI:** 10.1007/s00420-022-01922-z

**Published:** 2022-09-12

**Authors:** Krassi Rumchev, Dong Van Hoang, Andy H. Lee

**Affiliations:** 1 School of Public Health, Curtin University, Perth, Australia; 2 National Center for Global Health and Medicine, Tokyo, Japan

**Keywords:** Underground mines, Respiratory symptoms, Inhalable and respirable dust, Australia

## Abstract

**Purpose:**

Occupational exposure to dust has been recognised as a significant health hazard to mine workers. This study aimed to investigate the association between exposure to inhalable (INH) and respirable (RES) dust and respiratory health among mine workers in Western Australia using an industry-wide exposure database.

**Methods:**

The database comprised cross-sectional surveys conducted by mining companies for the period 2001–2012. The study population consisted of 12,797 workers who were monitored for exposure to INH and RES dust and undertook health assessments including a respiratory questionnaire and spirometry test.

**Results:**

Despite the general trend of declining exposure to both INH and RES dust observed over the 12 years period, mine workers reported a higher prevalence of phlegm and cough when exposed to elevated concentrations of INH and RES dust. Logistic regression analysis further confirmed the positive association between INH dust exposure and the prevalence of phlegm with an adjusted odds ratio of 1.033 (95% CI 1.012–1.052). Overall, 6.3% of miners might have potential airway obstruction, and exposure to INH dust was associated with impaired lung function parameters.

**Conclusion:**

Exposure levels of INH and RES dust particles among mine workers have reduced considerably and were well below currently legislated occupational exposure limits. However, given the reported higher prevalence of phlegm and cough among those with elevated dust concentrations, there is a continued need for effective dust exposure monitoring and control in the mineral mining industry.

## Introduction

Mining is commonly defined as the extraction of valuable minerals and other materials from the earth. This is considered one of the major economic activities that contribute to the advancement of economies worldwide. With the current advancement in technology and technological products, the demands for minerals are increasing and consequently the need for mining activities to meet these demands (Abraham 2015).

However, mine site activities involve significant hazards and risks associated with exposures due to different work processes and the generation or use of hazardous substances (ILO 2014; Donoghue 2004). These include explosions, fires, collapses, cave-ins, rockfalls and dust exposures (Pandey et al. 2017). As a result, mine workers are commonly exposed to fibres and dust with variable aerodynamic particle distribution, including inhalable (INH) and respirable (RES) particle fractions. The term dust is defined as airborne particles, usually in a size range from 1 to 100 μm (WHO 1999; ISO7708 1995). INH particulates, smaller than 100 μm, are that fraction of a dust cloud that can be breathed into the nose or mouth and often get trapped in the upper respiratory tract (WHO 1999). It is thus associated with respiratory disorders, such as asthma, tracheitis, pneumonia and allergic rhinitis (WHO 1999; Shekarian et al. 2021). RES particulates are that mass fraction of inhaled particles with a 50% cut-point of 4 μm (WHO 1999; ISO7708 1995), and can penetrate beyond the terminal bronchioles into the gas-exchange region of the lungs and enter the bloodstream, where they can affect internal organs (WHO 1999; EN 13205, 2014; Schatzel et al. 2012; Wippich et al. 2020).

Mine workers are exposed to dust because the whole process of extracting minerals involves rock breaking through blasting and drilling as well as loading and unloading materials (Onder et al. 2009). Exposure to dust can be short-term or long-term and can cause respiratory health problems ranging from acute to chronic (Mamuya et al. 2007; Nelson 2013; Nkrumah and Yaw, 2005). Indeed, health consequences due to dust exposure include respiratory symptoms, chronic bronchitis, silicosis, tuberculosis, emphysema, renal failure and cancer (Cecala et al. 2019; Shekarian et al. 2021). There is also significant evidence that shows an increasing effect of total cumulative dust exposure on breathlessness (Bio et al. 2007).

The Occupational Safety and Health Administration has a permissible exposure limit for RES dust not to exceed 5 mg/m^3^ over an 8-h time-weighted average limit for workplace exposures (NIOSH 2018). The American Conference of Governmental Industrial Hygienists has guidelines that recommend airborne concentrations of RES dust be kept below 3 mg/m^3^ and for INH dust be kept below 10 mg/m^3^. Australia has similarly established exposure limits for INH and RES dust in mineral mines of 10 mg/m^3^ and 3 mg/m^3^, respectively (Work Health and Safety (Mines) Regulations 2022). The workplace exposure limits are defined as airborne concentrations of a particular chemical or substance in the workers’ breathing zone that should not cause adverse health effects or cause undue discomfort to nearly all workers.

In the literature, the morbidity and mortality from exposure to dust among mine workers have been well described. However, there is still a lack of any contemporary scientific evidence regarding INH and RES particles from mine inspections over a long period. The objective of the present study was to summarise the recent trend of exposure to INH and RES dust and the associated respiratory health among mine workers by using 12 years of compliance sampling data collected in Australian mineral mines.

## Methods

### Study population

Mine sites in the State of Western Australia are required to electronically submit their atmospheric contaminant results to the State’s Department of Mines and Petroleum (DMP) to assess compliance with dust regulations. The requested number of samples by DMP is determined on the mine site. For practical reasons, miners with similar exposure profiles are grouped by the mining companies before samples were randomly selected from each similar exposure group. A ventilation officer or hygienist was appointed by each mining company to collate the required data, called CONTAM. The type of mines included in the present study consisted of gold, nickel, iron and other mineral mines.

The present study used the 2001–2012 dataset provided by DMP. During the period 2001–2012, a total of 61,482 Western Australian mine workers were monitored for exposures to INH and RES dust. Of this population, 12,797 distinct workers undertook health assessments which constituted our study sample of 13,170 observations as some workers were surveyed more than once during the 12 years. The study population was involved in 129 mining-related activities. To facilitate analysis, these activities were classified into three occupation groups, namely ‘managers’, ‘surface production and services’ and ‘underground mining’. The first group represented mine management occupations, including managers, operation supervisors, superintendents and engineers. The second group included occupations with limited exposure to dust-generating activities, such as geologists, mobile plant operators, electricians and mechanics. The third group comprised those directly involved in underground mining production activities, such as drilling, blasting and loading. The available dataset was summarised by calendar year, occupation, age, gender, work shift length and current smoking status (in the last three months).

### Assessment of exposure to INH and RES particles

The mining companies conducted measurements of personal exposure to RES and INH particles, but only 1062 (8.3%) workers were monitored for both RES and INH dust. Air sampling of RES dust was performed using SKC Plastic Cyclone that holds a collection filter in a reusable cassette according to the Australian Standard (*Workplace atmospheres – Method for sampling and gravimetric determination of respirable dust)* (AS 2985,2009). Personal exposure to INH dust was monitored using an IOM particle size-selective sampler following the Australian Standard (*Workplace atmospheres – Method for sampling and gravimetric determination of inhalable dust)* (AS 3640,2009).

### Health assessment

Mine workers were invited to take part in a health assessment. The health surveillance comprised a respiratory questionnaire, lung function test, an audiometric (hearing) test and in some cases, a chest X-ray. In this study, we were provided with data collected through a respiratory questionnaire and spirometry test which comprised the MineHealth database. The survey consisted of questions related to current respiratory symptoms experienced in the last three months, namely cough, phlegm, wheeze and breathlessness, and further details on the health data collection have been described previously (Rumchev et al., 2020). Some personal information was also collected, including gender, age, job title, shift length (10 h or 12 h) and current smoking status (yes or no). Spirometry tests were performed by a medical practitioner or approved person from the company to determine the forced vital capacity (FVC) and forced expiratory volume in 1 s (FEV1). The ratio FEV1/FVC was then calculated for each mine worker to determine his/her potential airway obstruction, about the guidelines and boundary values for Australian adults (Brazzael et al., 2016).

Mine companies were responsible for the collection of hygiene (CONTAM) and health data (MineHealth) which was then entered into the Safety Regulations System (SRS). The CONTAM and MineHealth database were linked, and records were matched by the DMP using each worker’s unique identifier number.

The authors were not involved in the data collection and therefore did not seek ethics approval. The data used in the study were compliance sampling data collected in Western Australian mineral mines requested by DMP.

### Statistical analysis

Our analysis was based on estimated current exposures to INH and RES, current respiratory symptoms (last three months) and lung function parameters. INH and RES particle concentrations were presented as geometric mean (GM) with geometric standard deviation (GSD) due to their skewed distributions. These geometric means were stratified by personal characteristics (gender, occupation, shift length, smoking status), and according to the presence/absence of respiratory symptoms (cough, phlegm, breathlessness, wheeze). The overall prevalence of each respiratory symptom was calculated and tabulated concerning personal characteristics. Wilcoxon sign test and Kruskal–Wallis test were used for comparison of geometric means across categories as appropriate, whereas the Chi-squared test was applied to compare the prevalence of respiratory symptoms between personal characteristic groups.

To ascertain the association between RES and INH exposure levels and the prevalence of respiratory symptoms, logistic regression models were fitted, with adjusted odds ratios (OR) and their corresponding 95% confidence intervals (CI) for quantifying the magnitude of the observed association, while accounting for the effects of plausible confounding personal factors particularly smoking status. Finally, multiple linear regression analyses were undertaken to assess the relationship between INH and RES exposures and lung function parameters. All statistical analyses were performed in RStudio for Windows (version 3.2.4) (RStudio Team 2012).

### Results

#### Personal characteristics

The mean age of the workers was 36 (SD 11.1) years. Males comprised 90.8% of the mine workers with nearly half (47.3%) being directly engaged in underground mining activities, followed by surface operations (32.5%) and managerial work (20.1%). A great majority (77.6%) of them worked longer shifts (12 h), while about two-thirds (66.4%) reported as non-smokers.

### Exposure to INH and RES particles

**Fig. 1:**
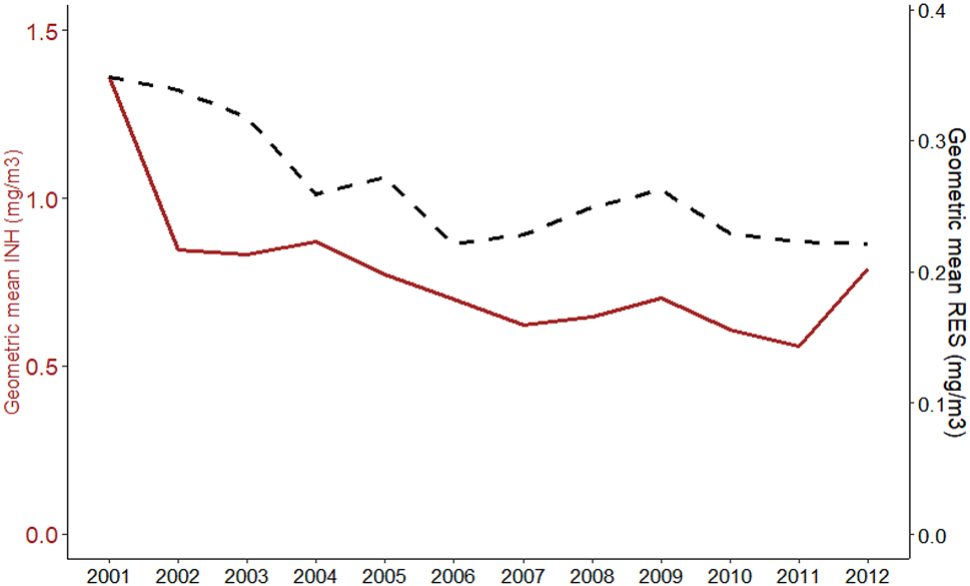
Geometric mean of INH and RES dust exposures among Australian miners, 2001–2012

In total, 8561 RES and 5683 INH dust samples were collected from the 12,797 mine workers with reported respiratory health during the period 2001–2012. Figure 1 presents the GM of these measurements, which exhibited a general declining trend over time (*p* < 0.001).

As shown in Table 1, the overall GM for INH (0.78 mg/m^3^) and RES (0.26 mg/m^3^) dust were well below the respective (Work Health and Safety (Mines) Regulations 2022) exposure limits of 10 mg/m^3^ and 3 mg/m^3^, with only 2.9% and 0.9% of the samples, respectively, exceeding the limits. Male workers were exposed to significantly higher INH and RES dust concentrations than their female counterparts. The study also found significantly higher mean RES and INH dust concentrations among mine workers who were current smokers when compared to non-smokers (Table 1).

**Table 1:** Exposure levels of INH and RES dust by personal characteristics among Australian miners

Characteristic	INH			RES		
	*N*	GM (GSD)	High exposure (%)^a^	*N*	GM (GSD)	High exposure (%)^b^
Overall	5683	0.78 (3.52)	167 (2.9)	8561	0.26 (2.69)	80 (0.9)
Gender, *n* (%)						
Female	533	0.51 (3.19)	10 (1.9)	772	0.22 (2.48)	4 (0.5)
Male	5150	0.75 (3.53)	157 (3.0)	7789	0.26 (2.70)	76 (1.0)
*P*^c^		< 0.001			< 0.001	
Occupation, *n* (%)						
Manager	1391	0.81 (3.45)	45 (3.2)	1546	0.26 (2.66)	13 (0.8)
Surface production	2182	0.76 (3.63)	69 (3.2)	2487	0.26 (2.77)	25 (1.0)
Underground mining	2110	0.64 (3.42)	53 (2.5)	4528	0.25 (2.65)	42 (0.9)
*P*^c^		< 0.001			0.569	
Shift length, *n* (%)						
10 h	1442	0.81 (3.70)	45 (3.1)	1763	0.26 (2.73)	17 (1.0)
12 h	4241	0.70 (3.45)	122 (2.9)	6798	0.25 (2.68)	63 (0.9)
*P*^c^		< 0.001			0.817	
Smoking status, *n* (%)						
Yes	1826	0.82 (3.47)	58 (3.2)	2927	0.28 (2.69)	30 (1.0)
No	3857	0.69 (3.53)	109 (2.8)	5634	0.25 (2.68)	50 (0.9)
*P*^c^		< 0.001			< 0.001	

*GM* geometric mean

^a^ > 10 mg/m^3^

^b^ > 5 mg/m^3^

^c^from Wilcoxon sign test or Kruskal–Wallis test

### Prevalence of respiratory symptoms

The most prevalent respiratory symptom was cough (15.1%), followed by wheeze (13.3%), phlegm (11.3%) and breathlessness (9.8%). Table 2 summarises the prevalence of respiratory symptoms by personal characteristics, which shows that male workers and those who worked at underground mines generally reported higher symptom rates. Moreover, current smokers sustained a significantly higher prevalence of respiratory symptoms when compared to their non-smoking colleagues.

**Table 2:** Prevalence of respiratory symptoms by personal characteristics among Australian miners

Characteristic	Respiratory symptom			
	Cough	Phlegm	Breathlessness	Wheeze
Gender, *n* (%)				
Female	149 (12.3)	85 (7.0)	122 (10.1)	136 (11.2)
Male	1759 (14.7)	1319 (11.0)	1098 (9.2)	1552 (13.0)
*P*^a^	0.025	0.001	0.337	0.089
Occupation, *n* (%)				
Manager	405 (15.3)	254 (9.6)	245 (9.2)	376 (14.2)
Surface production	595 (13.9)	417 (9.7)	379 (8.8)	568 (13.3)
Underground mining	908 (14.6)	733 (11.8)	596 (9.6)	744 (14.9)
*P*^a^	0.263	0.001	0.460	0.008
Shift length, *n* (%)				
10 h	431 (14.6)	342 (11.6)	297 (10.1)	411 (14.0)
12 h	1477 (14.4)	1062 (10.4)	923 (9.0)	1277 (12.5)
*P* ^a^	0.826	0.063	0.089	0.040
Smoking status, *n* (%)				
Yes	1189 (26.9)	821 (18.6)	550 (12.4)	875 (19.8)
No	719 (8.2)	583 (6.7)	670 (7.7)	813 (9.3)
*P*^a^	< 0.001	< 0.001	< 0.001	< 0.001

^a^from Chi-squared test

Exposure levels of INH and RES dust were next compared between miners according to their respiratory symptoms. The results showed that the 590 workers who reported phlegm were exposed to significantly higher GM concentrations of INH dust (0.90 µg/m^3^) compared with their asymptomatic colleagues (0.71 µg/m^3^) (*n* = 5093).

The logistic regression results, overall and stratified by smoking status, are summarised in Table 3, confirming the significant positive association between INH dust exposure and the prevalence of phlegm regardless of the smoking status of mine workers. According to the fitted overall models being a smoker adversely affected the respiratory health of mine workers, with a significant (*p* < 0.001) increase in the prevalence of all respiratory symptoms. Age was also positively and consistently associated with respiratory symptoms; however, the associations with gender, occupation and shift length were less apparent according to the multivariate models.

**Table 3:** Logistic regression analyses of respiratory symptoms in relation to dust exposure, overall and stratified by smoking status

Respiratory symptom	INH (µg/m^3^)		RES (µg/m^3^)	
	Adjusted OR (95% CI)^a^	*P*	Adjusted OR (95% CI)^a^	*P*
Overall				
Cough	1.007 (0.986, 1.027)	0.473	0.986 (0.935, 1.029)	0.562
Phlegm	1.033 (1.012, 1.052)	0.001	0.981 (0.922, 1.029)	0.494
Breathlessness	0.996 (0.968, 1.021)	0.773	0.925 (0.836, 0.997)	0.082
Wheeze	1.005 (0.983, 1.025)	0.664	0.969 (0.911, 1.017)	0.262
Smokers				
Cough	1.004 (0.977, 1.030)	0.752	1.015 (0.949, 1.078)	0.644
Phlegm	1.031 (1.004, 1.058)	0.021	0.976 (0.889, 1.051)	0.559
Breathlessness	0.999 (0.961, 1.033)	0.972	0.923 (0.793, 1.031)	0.232
Wheeze	1.010 (0.981, 1.038)	0.466	0.982 (0.898, 1.055)	0.648
Non-smokers				
Cough	1.013 (0.978, 1.042)	0.430	0.946 (0.841, 1.019)	0.251
Phlegm	1.034 (1.002, 1.062)	0.023	0.986 (0.901, 1.043)	0.696
Breathlessness	0.993 (0.949, 1.029)	0.729	0.925 (0.801, 1.013)	0.199
Wheeze	0.997 (0.960, 1.028)	0.845	0.961 (0.873, 1.022)	0.311

*OR* odds ratio, *CI* confidence interval

^a^Adjusted for age (years), gender (female, male), occupation (manager, surface production, underground mining), shift length (10 h, 12 h) and additional adjustment for smoking status (yes, no) in overall models

#### Lung function

Mean FEV1, FVC and FEV1/FVC of the sample population were 3.97L (SD 0.78), 4.87L (SD 0.94) and 0.82 (SD 0.07), respectively. Overall, 11.9%, 12.4% and 6.3% of the sample might be considered to have impaired lung function in reference to the respective boundary values for the three parameters, FEV1, FVC and FEV1/FVC, respectively. The results from linear regression analyses, overall and stratified by smoking status, are presented in Table 4. The overall models suggest that exposure to INH dust was negatively associated with the lung function parameters, albeit with small magnitudes for the effect size. On the other hand, exposure to RES had little association with the lung function of mine workers, after adjusting for the potential effects of personal factors. When stratified by smoking, the linear regression analyses demonstrated a significant impact on lung function only for those who reported current smoking.

**Table 4:** Multiple linear regression analyses of lung function in relation to dust exposure, overall and stratified by smoking status

Lung function	INH (µg/m^3^)		RES (µg/m^3^)	
	Coefficient (95% CI)^a^	*P*	Coefficient (95% CI)^a^	*P*
Overall				
FEV1	– 0.009 ( – 0.014, – 0.004)	< 0.001	0.002 ( – 0.006, 0.010)	0.647
FVC	– 0.006 ( – 0.011, – 0.002)	0.006	– 0.001 ( – 0.009, 0.007)	0.793
FEV1/FVC	– 0.002 ( – 0.005, 0.000)	0.077	0.003 ( – 0.001, 0.007)	0.165
Smokers				
FEV1	– 0.014 ( – 0.026, – 0.001)	0.028	0.007 ( – 0.008, 0.021)	0.352
FVC	– 0.023 ( – 0.038, – 0.007)	0.004	– 0.002 ( – 0.015, 0.010)	0.741
FEV1/FVC	0.001 ( – 0.001, 0.002)	0.480	– 0.006 ( – 0.016, 0.005)	0.291
Non-smokers				
FEV1	– 0.006 ( – 0.015, 0.003)	0.190	– 0.002 ( – 0.010, 0.005)	0.566
FVC	– 0.006 ( – 0.017, 0.005)	0.305	0.002 ( – 0.005, 0.009)	0.515
FEV1/FVC	– 0.000 ( – 0.001, 0.001)	0.590	– 0.003 ( – 0.011, 0.004)	0.393

*CI* confidence interval

^a^Adjusted for age (years), gender (female, male), occupation (manager, surface production, underground mining), shift length (10 h, 12 h) and additional adjustment for smoking status (yes, no) in overall models; dust concentration was square-root transformed before being fitted in the linear regression models

## Discussion

The present study provided a comprehensive investigation of INH and RES dust exposure and respiratory health in the Western Australian mineral mining industry. The assessment for the period 2001–2012 was derived from measurements that complied with the government dust regulations. A general trend of declining exposure to both INH and RES was observed over the 12 years. Overall, only 0.9% of the RES samples exceeded the exposure limit of 3 mg/m^3^. This can be explained by the companies’ effort to comply with the stringent occupational health and safety regulations imposed by the DMP in Western Australia. Our estimated GM concentration of RES (0.26 mg/m^3^) was the same as that measured from underground gold miners in 2003–2004 in Tanzania (Rusibamayila et al. 2018), and similar to the levels (0.4 mg/m^3^) observed at the underground gold mine in South Africa during the period 2015–2016 (Nkrumah and Yaw 2005). However, the observed dust exposure levels were lower than those reported from studies conducted in earlier periods, including a study from Ghana (2000) (0.83 mg/m^3^) (Bio et al. 2006), the United States (1970–1980)(1.1 mg/m^3^) (Kuempel et al. 2003), Australia (1985–1999) (1.51 mg/m^3^) (Kizil and Donoghue 2002) and South Africa (1985 and 1998) (1.9 mg/m^3^) (Naidoo et al. 2004).

Similarly, less than 3% of the INH samples exceeded the Australian exposure limit of 10 mg/m^3^, with the overall GM concentration being 0.78 mg/m^3^ and much lower than the 100 mg/m^3^ measured in German uranium mines from 1945 to 1985 (Dahmann et al. 2008). As can be expected, their recorded median RES concentration was also high at 20 mg/m^3^. These high levels of dust exposures were typically encountered in the early years, especially in the years before 1953 of dry drilling and low ventilation conditions (velocities below 0.1 m/s), which subsequently improved after the enforcement by governments under better technical and organisational circumstances. Indeed, our observed declining trend in dust exposures was consistent with previous reports worldwide (Mannetje et al. 2002).

This study established higher exposures to INH dust among managers (GM 0.81 mg/m^3^) and surface production mine workers (GM 0.76 mg/m^3^) compared with those measured for underground mine workers (GM 0.64 mg/m^3^). This unexpected finding is difficult to explain because the authors were not involved in the data collection. It is assumed that the UG workers monitored in this study worked in enclosed environments, such as tracks, excavators and boggers, and were better protected from dust exposure compared to those who worked in open spaces and in office environments, including managers and service workers. Dust particles can accidentally be introduced indoors from shoes, clothes and things that are carried inside (Licina and Nazaroff, 2018). Outdoor and vehicle exhaust particulates can also be blown inside through open windows and doors allowing the dust to accumulate to higher levels.

Significantly higher concentrations for INH and RES dust were measured for mine workers who reported current smoking. Cigarette smoking is known as a potent source of particulate matter (dust) (Apelberg et al. 2013; Repace and Lowrey, 1980). Invernizzi and colleagues (2004) showed that the particulate air pollution emitted by cigarettes is 10 times greater than diesel car exhaust. The study measured 88 µg/m^3^ of combined particulate levels in the first hour after the engine starts compared with 830 µg/m^3^ of those recorded in the first hour after the cigarettes had been lit measured which is almost 10 times greater.

Friedman et al. (2019) reported that miners usually modify their activities when working long hours. Mine workers on 12-h shifts might move to a less dust-generating activity after working long hours, which could explain their recorded lower levels of INH dust when compared with their counterparts on 10-h shifts.

It is well known that high exposure to occupational dust can lead to adverse respiratory health. The importance of measuring different dust fractions in work environments has also been acknowledged for the evaluation of exposure and risk to workers (Wippich et al. 2020). Previously, mainly INH dust was sampled with limited data on RES until the introduction of guideline values. The current study presented population data on INH and RES dust exposures in relation to respiratory health. Despite the consistently low levels of observed mine dust concentrations over the study period, the prevalence of cough was still high at 15.1%. Indeed, the prevalence of cough among underground mine workers (14.6%), surface production (13.9%) and managers (15.3%) were higher than the 12.5% reported by the general population (Abramson et al. 2002).

This study found no significant difference in the prevalence of respiratory symptoms between miners working 10- and 12-h shift except for wheezing. Again, miners on 12-h shift might move to a less dust-generating activity after working long hours, which lead to their reduced prevalence of wheeze than others on 10-h shift.

Moreover, our multivariate analyses confirmed that INH dust exposure was positively associated with the prevalence of phlegm, while being a smoker significantly increased the prevalence of all respiratory symptoms. The results are consistent with findings from studies conducted in Tanzania, Ghana and South Africa (Rusibamayila et al. 2018; Bio et al. 2007; Girdler Brown et al. 2008).

On the other hand, exposure to RES dust appeared to have little association with respiratory symptoms after accounting for the personal confounding factors. Seixas et al. (1992) concluded that RES dust concentration is a sensible proxy for measuring larger particles, while the development of some respiratory symptoms might be more closely related to larger dust particles, such as INH dust which is the case in the current study. Similar findings have also been reported elsewhere (Mamuya et al. 2007).

The American Thoracic Society and the European Respiratory Society provided guidelines for spirometry assessment (Ferguson et al. 2000; Pellegrino et al. 2005) and their reference values have been endorsed by various international respiratory societies, including the Thoracic Society of Australia and New Zealand (Brazzael et al. 2016). According to the guidelines, the reference value of < 0.70 recommended for the ratio FEV1/FVC is to define airway obstruction. In this study, despite the observed mean ratio of 0.82, 6.3% of the sample population of mine workers were considered to have impaired lung function (< 0.7). In addition, larger INH particles rather than RES dust exposures appeared to be related to the decrease in lung function parameters, again consistent with previous reports by Mamuya et al. (2007). In addition, from the stratified logistic regression analysis, it appeared that only mine workers who reported current smoking were adversely impacted by their exposure to INH dust. Our finding is consistent with previous studies that demonstrated increased susceptibility to adverse health impacts among smokers (Arcavi and Benowitz, 2004; Anderson 2014; Feldman and Anderson, 2013).

The major strength of this study was the use of a large population database with quantitative exposure measurements on INH and RES dust over an extended period of 12 years. However, the following limitations should be taken into account when interpreting the findings. The ‘healthy worker effect’ is a type of selection bias that typically arises in observational studies of occupational exposures (Shah 2009). It is related to the initial recruitment of mainly healthy workers and is acknowledged as a study limitation. The observations through cross-sectional surveys have made it difficult to establish causal inference concerning dust exposure and respiratory health, especially since the data were collected for compliance purposes in accordance with DMP requirements, even though the same approach has been adopted elsewhere in occupational dust exposure assessments (Lavoué et al. 2005; Burstyn et al. 2000; Peters et al. 2011; Doney et al. 2020). The reliance on self-reported respiratory symptoms without medical diagnoses posed another limitation. Nevertheless, self-report data from the respiratory health survey had been used in conjunction with the quantitative INH and RES measurements and other information through record matching and data linkage to reduce bias and inaccuracy (Althubaiti 2016). Residual confounding was also a concern, with length and level of dust exposure, as well as information on other potential factors not captured in the database, which could affect the association between the prevalence of respiratory symptoms and exposure to INH and RES dust.

Finally, spirometry tests have been commonly used for assessing lung function among mine workers (Lin et al. 2008; Swanney et al. 2008). However, it is acknowledged that spirometry tests cannot identify lung disease at an early stage. In addition, until 2019 there were no standards for spirometry testing of lung function for mine workers in Australia. Therefore, there might have been inconsistency in performing the spirometry tests during the study period and this is acknowledged as a study limitation. Furthermore, applying a fixed FEV1/FVC cut-off of less than 0.7 could lead to under-diagnosis of airway obstruction (false negatives) in the younger population and over-diagnosis of airway obstruction (false positives) in the older population (Brazzael et al., 2016). Nevertheless, the simplicity of this measure overshadows its disadvantage, and it is a common practice for assessing lung function among mine workers (Lin et al. 2008; Swanney et al. 2008). Despite these limitations, the present study provides data for the best available exposure proxy for the mining industry in Western Australia and is informative for assessing trends in inspector‐reported INH and RES dust levels in associations with respiratory health among mine workers.

## Conclusion

This study demonstrated that mine workers still reported a higher prevalence of phlegm and cough when exposed to elevated concentrations of INH and RES dust. The findings underscore the continued need for effective dust exposure monitoring and control for all miners. In addition, it is recommended that mining companies should conduct awareness campaigns on the adverse effects of cigarette smoking on respiratory health.

## Data Availability

The datasets analysed during the current study are available from the corresponding author on reasonable request.
