# Correction: Exposure to dust and respiratory health among Australian miners

**DOI:** 10.1007/s00420-023-01957-w

**Published:** 2023-02-01

**Authors:** Krassi Rumchev, Dong Van Hoang, Andy H. Lee

**Affiliations:** 1 School of Public Health, Curtin University, Perth, Australia; 2 National Center for Global Health and Medicine, Tokyo, Japan

**Correction: International Archives of Occupational and Environmental Health** 10.1007/s00420-022-01922-z

Unfortunately the reference was wrongly published. The correct reference is given below.

1. Work Health and Safety (Mines) Regulations 2022, Available online: https://legislation.wa.gov.au/.
2. Exposure limits for dust in mineral mines, Available online: https://www.business.qld.gov.au/industries/mining-energy-water/resources/safety-health/mining/hazards/dust/exposure-limits.

